# Assessment of Opioid Prescribing Patterns in a Large Network of US Community Health Centers, 2009 to 2018

**DOI:** 10.1001/jamanetworkopen.2020.13431

**Published:** 2020-09-18

**Authors:** John Muench, Katie Fankhauser, Robert W. Voss, Nathalie Huguet, Daniel M. Hartung, Jean O’Malley, Steffani R. Bailey, Stuart Cowburn, Dagan Wright, Gordon Barker, Maria Ukhanova, Irina Chamine

**Affiliations:** 1 Department of Family Medicine, Oregon Health & Science University, Portland; 2 OCHIN Inc, Portland, Oregon; 3 Department of Pharmacy Practice, Oregon State University, Portland; 4 Department of Public Health and Preventive Medicine, Oregon Health & Science University, Portland

## Abstract

**Importance:**

Understanding opioid prescribing patterns in community health centers (CHCs) that disproportionately serve low-income patients may help to guide strategies to reduce opioid-related harms.

**Objective:**

To assess opioid prescribing patterns between January 1, 2009, and December 31, 2018, in a network of safety-net clinics serving high-risk patients.

**Design, Setting, and Participants:**

Cross-sectional study of 3 227 459 opioid prescriptions abstracted from the electronic health records of 2 129 097 unique primary care patients treated from 2009 through 2018 at a network of CHCs that included 449 clinic sites in 17 states. All age groups were included in the analysis.

**Main Outcomes and Measures:**

The following measures were described at the population level for each study year: (1) percentage of patients with at least 1 prescription for an opioid by age and sex, (2) number of opioid prescriptions per 100 patients, (3) number of long-acting opioid prescriptions per 100 patients, (4) mean annual morphine milligram equivalents (MMEs) per patient, (5) mean MME per prescription, (6) number of chronic opioid users, and (7) mean of high-dose opioid users.

**Results:**

The study population included 2 129 097 patients (1 158 413 women [54.4%]) with a mean (SD) age of 32.2 (21.1) years and a total of 3 227 459 opioid prescriptions. The percentage of patients receiving at least 1 opioid prescription in a calendar year declined 67.4% from 15.9% in 2009 to 5.2% in 2018. Over the 10-year study period, a greater percentage of women received a prescription (13.1%) compared with men (10.9%), and a greater percentage of non-Hispanic White patients (18.1%) received an opioid prescription compared with non-Hispanic Black patients (9.5%), non-Hispanic patients who self-identified as other races (8.0%), and Hispanic patients (6.9%). The number of opioid prescriptions for every 100 patients decreased 73.7% from 110.8 in 2009 to 29.1 in 2018. The number of long-acting opioids for every 100 patients decreased 85.5% during the same period, from 22.0 to 3.2. The MMEs per patient decreased from 1682.7 in 2009 to 243.1 in 2018, a decline of 85.6%.

**Conclusions and Relevance:**

In this cross-sectional study, the opioid prescribing rate in 2009 in the CHC network was higher than national population estimates but began to decline earlier and more precipitously. This finding likely reflects harm mitigation policies and efforts at federal, state, and clinic levels and strong clinical quality improvement strategies within the CHCs.

## Key Points

**Question:**

Are there prescribing patterns for opioid medications in safety-net clinics?

**Findings:**

In this cross-sectional study of 3 227 459 opioid prescriptions abstracted from electronic health records of patients treated at a network of community health centers between January 1, 2009, and December 31, 2018, the number of prescriptions per 100 primary care patients decreased 73.7% from 110.8 in 2009 to 29.1 in 2018. Other metrics of opioid use decreased to a similar degree.

**Meaning:**

Opioid prescribing in a large network of community health centers decreased from January 1, 2009, to December 31, 2018, which likely reflects a mix of federal, state, and local policies and initiatives directed at reducing harms associated with increased opioid prescribing in the early 2000s.

## Teaser

This cross-sectional study evaluates opioid prescribing patterns from 2009 to 2018 in a network of community health centers that frequently treat low-income patients in the US.

## Introduction

Opioid-related morbidity and mortality in the US have been well documented.^1,2^ This problem began with and continues to be propelled by opioid prescriptions from medical settings, with nearly half of these prescriptions generated from primary care clinics.^3,4^ Although opioid prescribing has declined steadily since 2011, prescription rates remain historically high with enough opioids dispensed for 1 in every 2 individuals in the US in 2018.^5^

Although the opioid epidemic affects all demographic groups, individuals from low-income communities are at highest risk for opioid-related adverse outcomes, including hospitalization and fatal overdose.^5,6,7,8^ Despite this increased risk, less is known about prescribing patterns in these communities. Most studies that have described prescribing trends relied on insurance claims data from managed care systems and retail pharmacies,^9,10,11,12,13,14^ sources that are likely to undercount unemployed and uninsured individuals, precisely those individuals most likely to have opioid-related adverse outcomes. Nationally, community health centers (CHCs) provide a disproportionate amount of care to the poor, the publicly insured, and the uninsured.^15^ As a consequence, CHC data are likely an ideal source of information concerning opioid prescribing patterns in low-income populations.

The aims of this study were to assess opioid prescribing patterns overall and stratified by patient demographic characteristics using electronic health record (EHR) data from a large network of CHCs from 2009 to 2018 and to explore how these patterns might differ from national population opioid prescription patterns.

## Methods

This study was approved by the Oregon Health & Science University Institutional Review Board. The institutional review board waived the requirement for patient informed consent because only deidentified data were used. This study adhered to the Strengthening the Reporting of Observational Studies in Epidemiology (STROBE) reporting guideline for cross-sectional studies.

### Data Sources, Study Design, and Population

We abstracted data from the OCHIN (not an acronym) network’s ambulatory EHR between January 1, 2009 (when OCHIN implemented a defined vocabulary system for prescriptions), and December 31, 2018. OCHIN is the nation’s largest network of CHCs (federally qualified health centers, county health department clinics, and not-for-profit clinics), including 449 clinics in 17 states (California, Oregon, Washington, Ohio, Wisconsin, Alaska, North Carolina, Indiana, Minnesota, Montana, Nevada, Texas, Massachusetts, Florida, Utah, Georgia, and New Mexico), that provide care to all individuals regardless of insurance status. The OCHIN clinics share a common EHR (EPIC; EPIC Systems Corporation) that facilitated data retrieval from across health systems and states.

### 

We summarized each study year separately, creating 10 cross-sectional assessments of prescribing patterns within OCHIN clinics. For each of the years in the 10-year study, we included all patients who had at least 1 visit with a primary care clinician at an ambulatory clinic during the respective calendar year (Table 1, eTable 1, and the eFigure in the Supplement). All age groups were included.

**Table 1.** Geographic Distribution of OCHIN Study Population Over Time

Year	No. (%)			
	California	Oregon	Other states^a^	All states^b^
2009	49 659 (33.00)	89 446 (59.40)	11 390 (7.60)	150 495 (100.00)
2010	84 417 (36.30)	125 715 (54.00)	22 664 (9.70)	232 796 (100.00)
2011	112 093 (32.80)	164 734 (48.20)	64 598 (18.90)	341 425 (100.00)
2012	125 098 (29.30)	195 869 (45.90)	106 005 (24.80)	426 972 (100.00)
2013	142 203 (28.60)	215 192 (43.30)	139 696 (28.10)	497 091 (100.00)
2014	198 577 (33.30)	233 913 (39.20)	164 576 (27.60)	597 066 (100.00)
2015	256 757 (36.00)	239 403 (33.50)	217 412 (30.50)	713 572 (100.00)
2016	263 860 (32.20)	238 346 (29.10)	316 931 (38.70)	819 137 (100.00)
2017	274 300 (31.90)	231 659 (27.00)	353 592 (41.10)	859 551 (100.00)
2018	269 658 (32.50)	218 102 (26.30)	343 101 (41.30)	830 861 (100.00)

^a^ Number of states included in this column changed with time (eTable 1 and the eFigure in the Supplement).

^b^ eTable 1 in the Supplement lists the included states for each year.

### Definitions and Variables

Medication prescribing records contain EPIC-generated medication IDs in addition to text fields containing the medication name, generic name, form, and pharmaceutical class. We searched the generic name field for all opioid medications that can be ordered with a prescription in outpatient settings in the United States (eTable 2 in the Supplement). We included medications with a pharmaceutical class of analgesic and excluded those with class of expectorant, antitussive, antidiarrheal, and all that were not oral or transdermal in form. We excluded buprenorphine with the exception of 2 forms that have been approved by the US Food and Drug Administration for pain (Butrans and Belbuca). Prescription order data included the ordering date, patient ID, prescriber ID, medicine name, strength (eg, 5 mg), number of units (eg, 30 tablets), and number of authorized refills. Refills were counted in the same year or quarter as the initiating prescription. We defined long-acting formulations as those that included the terms *extended release* or *long acting* in their generic name or form fields as well as methadone tablets and transdermal formulations. Using the Centers for Disease Control and Prevention’s (CDC’s) conversion reference table, ^16^ we calculated the morphine milligram equivalents (MMEs) for each prescription as the product of the medication strength, number of units, number of refills, and the relevant conversion factor.

Using the cohort year as the unit of analysis, we calculated several measures of opioid prescribing for each year of the study: (1) percentage of patients who received at least 1 prescription for an opioid by age and sex, (2) number of opioid prescriptions per 100 patients, (3) number of long-acting opioid prescriptions per 100 patients, (4) mean annual MME per patient, (5) mean MME per prescription, (6) number of chronic opioid users, and (7) number of high-dose opioid users. We defined chronic opioid users as patients who were prescribed within any calendar quarter 160 or more opioid pills (short acting or long acting), 90 or more long-acting pills, or any methadone pills or fentanyl patches. Prescription instructions were found to be unreliable in EHR data because many were composed of free text that could not be categorized, complicating the calculation of a daily dose. Therefore, we defined high-dose users as those individuals among chronic opioid users with a mean of more than 90 MMEs/d through any quarter of a calendar year.

Patient health insurance status and income relative to the federal poverty level (FPL) were defined at the patient’s index visit in the study period. Several of our parameters were designed to duplicate those in the CDC 2019 Annual Surveillance Report of Drug-Related Risks and Outcomes^5^ and a recent description of trends in the Veterans Health Administration (VHA).^17^

### Statistical Analysis

We used descriptive statistics to summarize the overall patterns and patterns stratified by age and sex in opioid prescribing at CHCs in 17 states for each year from 2009 to 2018 among the OCHIN primary care population. Analysis was conducted using R, version 3.6.0 (R Foundation for Statistical Computing).

## Results

Our study population consisted of 2 129 097 patients, including 1 158 413 female patients (54.4%) (mean [SD] age, 32.2 [21.1] years). A total of 3 227 459 opioid prescriptions were abstracted from the electronic health records. From 2009 to 2018, the OCHIN network grew from 150 495 patients in 3 states (California, Oregon, and Washington) to 830 861 patients in 15 states (Alaska, California, Georgia, Indiana, Massachusetts, Minnesota, Montana, North Carolina, New Mexico, Nevada, Ohio, Oregon, Texas, Washington, and Wisconsin) (Table 1 and eTable 1 in the Supplement). Demographic characteristics of the study population are summarized in Table 2. Over the 10-year study period, a total of 2 129 097 unique patients were included, of whom 257 848 (12.1%) had received 1 or more opioid prescriptions. The percentage receiving an opioid prescription was higher among women (13.1%) than men (10.9%). Non-Hispanic White patients received far more opioid orders (18.1%) than non-Hispanic Black patients (9.5%), non-Hispanic patients who self-identified as other races (8.0%), and Hispanic patients (6.9%), which supports previous reports.^18^ Similarly, there were fewer Spanish-speaking individuals with an opioid prescription (6.7%) compared with English-speaking individuals (14.0%). Uninsured patients had higher rates of receiving opioid prescriptions (13.5%) vs those who were publicly or privately insured (both 11.7%). The rate of opioid use did not differ by income categories, with 12.5% of individuals with an income greater than 138% of the FPL and 12.6% of individuals with an income less than or equal to 138% of the FPL.

**Table 2.** Patient Demographic Characteristics

Total	No. (%)		% Of category receiving an opioid
	OCHIN primary care patients, 2009-2018	Distribution of OCHIN patients with an opioid order	
No. (%)	2 129 097 (100)	257 848 (100)	12.1
Sex			
Female	1 158 413 (54.4)	151 738 (58.8)	13.1
Male	968 930 (45.5)	105 927 (41.1)	10.9
Other^a^	1754 (0.1)	183 (0.1)	10.4
Income relative to FPL^b^			
≤138%	1 260 571 (59.2)	158 658 (61.5)	12.6
>138%	269 627 (12.7)	33 722 (13.1)	12.5
Unknown	598 899 (28.1)	65 468 (25.4)	10.9
Race/ethnicity^c^			
Hispanic	660 909 (31.0)	45 820 (17.8)	6.9
Non-Hispanic			
White	877 355 (41.2)	158 693 (61.5)	18.1
Black	321 599 (15.1)	30 654 (11.9)	9.5
Other	107 103 (5.0)	8563 (3.3)	8.0
Unknown	162 131 (7.6)	14 118 (5.5)	8.7
Type of insurance^d^			
Private	301 994 (14.2)	35 436 (13.7)	11.7
Public	1 236 875 (58.1)	144 603 (56.1)	11.7
Uninsured	474 708 (22.3)	64 254 (24.9)	13.5
Unknown	115 520 (5.4)	13 555 (5.3)	11.7
Primary language			
English	1 560 317 (73.3)	217 773 (84.5)	14
Spanish	407 867 (19.2)	27 449 (10.6)	6.7
Other	117 978 (5.5)	9003 (3.5)	7.6
Unknown	42 935 (2.0)	3623 (1.4)	8.4

Abbreviation: FPL, federal poverty level.

^a^ Unknown or nonbinary sex.

^b^ FPL was taken from patient's first encounter, where non-null, during study period.

^c^ Patient-reported race and ethnicity. Patients identified as Hispanic if they self-reported being Hispanic in the electronic health record or reported Spanish as their primary language.

^d^ Type of health insurance was taken from a patient’s first encounter during study period.

Between 2009 and 2018, there was a 67.4% decline in the proportion of OCHIN patients of all ages who received an opioid prescription, with 15.9% receiving an opioid prescription in 2009 vs 5.2% receiving such a prescription in 2018 (Table 3). This rapid decline varied by age, with the largest decline occurring for patients younger than 15 years (−93.8%) and the smallest occurring for those 65 years or older (−54.7%). For those older than 19 years, the rate of decline was 42% between 2012 and 2016 alone. Although rates of decline were similar between male and female patients overall (−67.7% and −67.0%, respectively), the rates differed by age group. In particular, the rates of decline among female patients were greater than in male patients who were 65 years or older (−55.9% vs −51.7% respectively) and for those in the 20- to24-year age group (−83.5% vs −78.8%).

**Table 3.** Percent of OCHIN Patients With an Opioid Prescription by Age and Sex^a^

Age group, y	Sex	2009	2010	2011	2012	2013	2014	2015	2016	2017	2018	% Change, 2018 vs 2009
0-14	Total	0.67	0.50	0.48	0.43	0.52	0.37	0.23	0.14	0.09	0.04	−93.8
	Male	0.68	0.46	0.45	0.41	0.53	0.39	0.22	0.15	0.10	0.04	−94.6
	Female	0.66	0.54	0.52	0.44	0.50	0.35	0.24	0.14	0.09	0.05	−92.9
15-19	Total	3.7	4.1	3.5	3.0	3.4	2.5	1.7	1.2	0.83	0.51	−86.5
	Male	3.3	3.9	2.9	3.0	3.4	2.0	1.6	1.1	0.75	0.46	−86.3
	Female	4.0	4.3	3.9	3.1	3.4	2.8	1.8	1.3	0.90	0.54	−86.3
20-24	Total	10.1	10.7	9.4	8.4	8.6	6.9	5.2	3.6	2.7	1.8	−82.3
	Male	8.7	10.4	9.4	9.0	8.8	7.1	5.0	3.5	2.7	1.8	−78.8
	Female	10.7	10.8	9.4	8.2	8.5	6.9	5.2	3.6	2.8	1.8	−83.5
25-34	Total	16.2	15.9	14.8	13.3	13.3	11.6	8.8	6.6	5.1	3.7	−77.3
	Male	16.4	15.9	15.5	13.8	13.9	11.7	8.5	6.6	4.9	3.4	−79.0
	Female	16.1	15.8	14.4	13.0	12.9	11.5	8.9	6.7	5.2	3.8	−76.3
35-44	Total	23.0	22.0	19.1	17.8	17.6	15.6	12.3	9.4	7.4	5.6	−75.4
	Male	22.4	22.0	18.6	17.6	17.5	15.2	11.9	9.0	7.1	5.3	−76.1
	Female	23.4	21.9	19.4	17.9	17.6	15.9	12.6	9.7	7.6	5.8	−75.0
45-54	Total	31.2	29.9	26.3	24.1	23.4	20.8	16.7	13.2	10.6	8.3	−73.3
	Male	30.2	29.1	25.4	23.5	22.5	19.9	15.7	12.4	10.0	7.9	−73.8
	Female	32.0	30.5	26.9	24.5	24.0	21.5	17.4	13.8	11.0	8.7	−72.9
55-64	Total	30.9	29.1	26.8	25.5	25.1	22.7	19.1	16.1	13.3	11.3	−63.4
	Male	30.2	28.0	25.8	24.9	24.4	22.0	18.6	15.6	12.8	10.7	−64.6
	Female	31.5	29.9	27.6	26.0	25.6	23.2	19.5	16.5	13.6	11.8	−62.4
≥65	Total	26.9	25.4	24.6	23.8	24.1	23.0	19.8	16.2	13.3	12.2	−54.7
	Male	22.4	21.3	21.0	20.5	20.8	19.9	17.5	14.7	12.2	10.8	−51.7
	Female	29.8	28.1	27.0	26.0	26.4	25.1	21.3	17.2	14.1	13.1	−55.9
All ages	Total	15.9	15.2	14.1	13.5	13.6	12.2	10.0	8.0	6.5	5.2	−67.4
	Male	14.7	14.0	12.9	12.5	12.6	11.2	9.1	7.3	6.0	4.7	−67.7
	Female	16.8	16.1	15.0	14.2	14.3	13.0	10.6	8.4	6.9	5.5	−67.0

^a^ Age group total rows include patients with unknown or nonbinary sex.

As shown in Table 4, the number of prescriptions per 100 patients decreased by 73.7% (from 110.8 in 2009 to 29.1 in 2018). Similarly, the number of long-acting opioid prescriptions per 100 patients declined from 22.0 in 2009 to 3.2 in 2018 (85.5% decline). A mean of 243.1 MMEs were prescribed for each OCHIN patient in calendar year 2018, an 85.6% decline from 1682.7 MMEs in 2009. Moreover, the mean MME per opioid prescription steadily decreased 50.4% from 1759.0 in 2009 to 873.3 in 2018. Chronic opioid users represented 8.1% of all OCHIN patients in 2009 and 1.9% of all OCHIN patients in 2018. In the same period, the proportion of high-dose users diminished from 2.2% to 0.3%.

**Table 4.** Trends in Opioid Prescribing

Year	No. of prescriptions per 100 primary care patients		MME		Opioid use among OCHIN primary care patients, No. (%)	
	Any analgesic opioid	Long-acting analgesic opioids	Per person annually	Mean per prescription (95% CI)	Chronic users	High-dose users
2009	110.8	22.0	1682.7	1759.0 (1743.6-1774.4)	12 193 (8.1)	3368 (2.2)
2010	110.4	20.4	1691.7	1793.7 (1780.5-1806.9)	17 580 (7.6)	4659.0 (2.0)
2011	96.1	16.3	1388.5	1692.8 (1681.7-1703.8)	23 654 (6.9)	5491.0 (1.6)
2012	85.7	13.7	1144.0	1560.0 (1550.6-1569.4)	26 794 (6.3)	5691.0 (1.3)
2013	84.6	12.0	1016.7	1406.0 (1397.9-1414.1)	29 735 (6.0)	5815.0 (1.2)
2014	72.3	9.1	783.1	1219.4 (1212.8-1226)	31 201 (5.2)	5506.0 (0.9)
2015	51.3	6.4	520.6	1071.8 (1066.3-1077.4)	26 457 (3.7)	4348.0 (0.6)
2016	42.5	5.1	406.8	1007.8 (1002.8-1012.8)	24 026 (2.9)	3688.0 (0.5)
2017	35.0	4.1	313.5	940.5 (935.7-945.3)	20 809 (2.4)	3182.0 (0.4)
2018	29.1	3.2	243.1	873.3 (868.7-878)	15 985 (1.9)	2250.0 (0.3)

Abbreviation: MME, morphine milligram equivalent.

## Discussion

Opioid prescribing in a large network of CHCs decreased between 2009 and 2018. In 2009, the national opioid problem was being recognized as an epidemic.^19,20^ At that time, the OCHIN system of safety-net clinics had rates of opioid prescribing that were as high as or higher than the CDC’s national estimates and the VHA population estimates (Figure). Nationally, it was estimated that 79.5 opioid prescriptions were written for every 100 Americans in 2009, totaling 733 MMEs per capita.^5^ In the OCHIN population, 110.8 opioid prescriptions were written per 100 patients, with 1682 MMEs per person for the same year.

**Figure.**
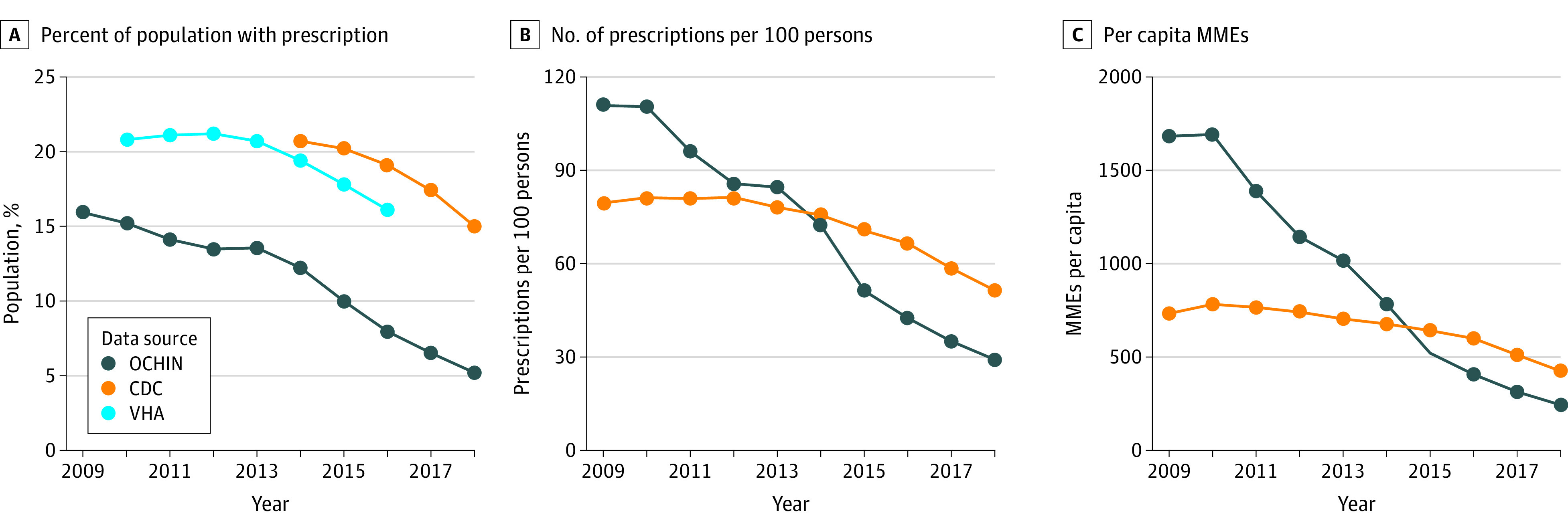
OCHIN and National Opioid Annual Prescribing Trends CDC indicates Centers for Disease Control and Prevention; MMEs, morphine milligram equivalents; VHA, Veterans Health Administration.

OCHIN prescribing rates peaked earlier and subsequently declined faster than most other epidemiologic descriptions of opioid prescribing. In this study, most OCHIN opioid prescribing measures had peaked at least by 2009, the first year for which data were available, the only exception being the MME per capita, which plateaued in 2009-2010 and declined thereafter. Nationally, most prescribing parameters peaked in 2012.^5,21^ A similar analysis of the VHA system demonstrated that opioid prescribing peaked in 2012 with subsequent decline.^17^ It should be noted that all of these measures show opioid prescribing was decreasing well before the CDC’s prescribing guidelines were promulgated in 2016.^22,23^

Similar to the CDC national estimates, female patients received more prescription opioids than male patients across nearly all age groups, although the magnitude of the difference in the OCHIN population was not as great as in national data. Previous studies have shown that patients with Medicaid^13,24,25^ and Medicare^26^ insurance are prescribed more opioids than those with commercial insurance. Within the CHC network, however, although the uninsured received more opioid prescriptions, little difference was found between those with public vs private insurance. Nor was a difference found between those with an income level that was 138% higher or lower than the FPL. Given that CHC populations are greatly weighted to publicly and uninsured individuals with far fewer commercially insured individuals (only 14.2% in this study), it is likely that patients with commercial insurance played a smaller role in the prescribing habits of clinicians.

The most marked observation in comparing OCHIN opioid prescribing patterns with other descriptions of prescribing patterns is the steep downward trajectory in OCHIN clinics. From their peaks in 2009 (OCHIN) and 2012 (national estimate^5^) through 2018, the number of prescriptions per 100 OCHIN patients declined 73.7% compared with 36.7% nationally.^5^ Similarly, between 2010 and 2018, OCHIN MMEs per capita declined 85.6%, whereas national trends decreased by 44.4%.^5^ Estimates show that the percentage of VHA patients receiving an opioid prescription decreased 24% (21.2% [2012] to 16.1% [2016]) between 2012 and 2016^17^; in the same years, OCHIN rates for those older than 19 years old decreased 42%.

Several hypotheses might explain the precipitous decline in prescribing rates in OCHIN CHCs. As in all populations, the decline in prescribing rates reflects federal, state, and local efforts to mitigate the harms from increased prescribing that had become apparent by 2009,^27,28^ with depictions of opioid harms becoming more prevalent in the national media.^29^

The difference may also reflect the nature of our national system of CHCs. Patient populations at CHCs generally have more physical and mental health comorbidity^15,30,31^ and have a lower income compared with the general population.^15^ Despite this complexity, CHCs surpass private clinics in many quality metrics,^15,32,33^ and there may be a heightened clinician awareness and attention to opioid quality initiatives in CHCs compared with private clinics. For example, in April 2011 the medical director of the Health Department of Multnomah County (MCHD) in Oregon became concerned that their clinical opioid prescribing rates might be contributing to new state public health data demonstrating rising opioid overdose and mortality (personal communication with Amit Shah, MD, January 24, 2020, medical director of MCHD in 2011). The MCHD leadership team developed several criteria for ascertaining when harm of opioid use might outweigh benefit, and required that records of patients meeting these criteria be reviewed by an opioid oversight committee. The criteria included, but were not limited to very high doses, coprescribing with benzodiazepine drugs, aberrant medication behavior, and suicidality. More than 400 cases were reviewed, and advice was communicated to all clinicians in the county health department, with a subsequent decrease found in their opioid prescribing rates.

Another possible explanation is that economically disadvantaged patients are less empowered to resist clinician-initiated tapers in their opioid dosing. A recent analysis of Medical Expenditure Panel Survey data by Olfson et al^34^ found that individuals with an income level lower than 100% of the FPL had the fastest decrease in opioid use from 2014 through 2016 compared with other income groups.

### Limitations

This study has limitations. First, the EHR data contain orders for prescriptions, and it is not possible to ascertain whether these orders were picked up by patients; therefore, the actual use of the opioids may have been overestimated. Second, the OCHIN population is disproportionately represented by West Coast states and may not be characteristic of all national CHCs or overall national population estimates. Third, the inclusion criteria required patients to have had an ambulatory encounter in the years they were counted, creating potential denominator differences compared with national population estimates, but this criterion is similar to that of a VHA study.^17^ Similarly, because of growth in the OCHIN network, there may have been changes in the study population denominator that biased our findings. For example, if patients added in later years were at lower risk for opioid use, this might have contributed to the prescribing declines that we observed.

## Conclusions

This retrospective, longitudinal, cross-sectional study of opioid prescribing in a large network of CHCs found an early and sharp reduction in opioid prescribing despite the greater complexity and lower income of CHC populations. These declines likely reflect earlier local efforts and clinical quality initiatives in CHCs to mitigate harms from overprescribing opioids. Further studies are needed to determine what specific policies and measures were most effective in these efforts.

## Notes

Supplement. **eTable 1. **Representation of states other than California and Oregon by year **eFigure.** Prescribing metrics distinguished by Oregon, California, and Other States **eTable 2. **List of included analgesic opioids with number and percentage
