# Supplementary material for: Assessment of Opioid Prescribing Patterns in a Large Network of US Community Health Centers, 2009 to 2018
Source: JAMA Netw Open. 2020 Sep 18;3(9):e2013431. doi: 10.1001/jamanetworkopen.2020.13431 (PMC7501536; doi:10.1001/jamanetworkopen.2020.13431)
Supplement: Supplement. — eTable 1. Representation of states other than California and Oregon by year eFigure. Prescribing metrics distinguished by Oregon, California, and Other States eTable 2. List of included analgesic opioids with number and percentage [file jamanetwopen-e2013431-s001.pdf]

Supplementary Online Content

Muench J, Fankhauser K, Voss RW, et al. Assessment of opioid prescribing patterns in a large network of US community health centers, 2009 to 2018. *JAMA Netw Open*. 2020;3(9):e2013431. doi:10.1001/jamanetworkopen.2020.13431

eTable 1. Representation of states other than California and Oregon by year
eFigure. Prescribing metrics distinguished by Oregon, California, and Other States
eTable 2. List of included analgesic opioids with number and percentage

This supplementary material has been provided by the authors to give readers additional information about their work.

© 2020 Muench J et al. *JAMA Network Open.*

eTable 1. Representation of states other than California and Oregon by year

Year	Other States
2009	WA
2010	OH WA WI
2011	AK NC OH WA WI
2012	AK IN MN MT NC NV OH TX WA WI
2013	AK IN MA MN MT NC NV OH TX WA WI
2014	AK FL IN MA MN MT NC NM NV OH TX UT WA WI
2015	AK FL GA IN MA MN MT NC NM NV OH TX UT WA WI
2016	AK FL GA IN MA MN MT NC NM NV OH TX UT WA WI
2017	AK FL GA IN MA MN MT NC NM NV OH TX UT WA WI
2018	AK GA IN MA MN MT NC NM NV OH TX WA WI

© 2020 Muench J et al. *JAMA Network Open.*

eFigure. Prescribing metrics distinguished by Oregon, California, and Other States

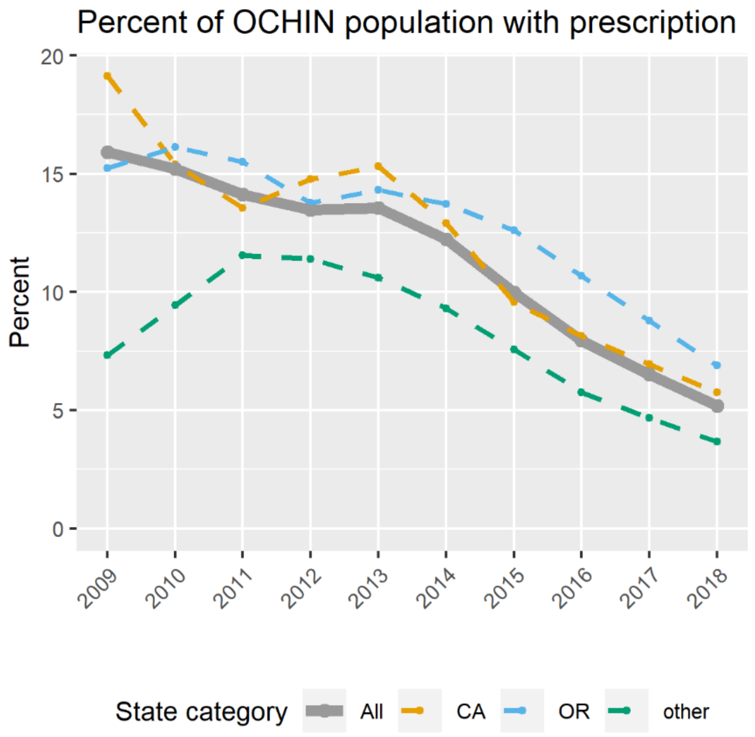

© 2020 Muench J et al. *JAMA Network Open.*

eTable 2. List of included analgesic opioids with numbers and percentages

Opioid Medication	n	%
Buprenorphine	3989	0.14
Codeine	89044	3.22
Fentanyl	53885	1.95
Hydrocodone	1121730	40.59
Hydromorphone	34454	1.25
Levorphanol	47	0
Meperidine	858	0.03
Methadone	154979	5.61
Morphine	224359	8.12
Oxycodone	726493	26.29
Oxymorphone	1715	0.06
Pentazocine	272	0.01
Propoxyphene	2576	0.09
Tapentadol	832	0.03
Tramadol	348200	12.6

© 2020 Muench J et al. *JAMA Network Open.*
